# Unexpected Predictors of Mortality During a DENV-3 Outbreak in Western Mexico: Seizures, Polyserositis, and Renal Dysfunction Without Severe Thrombocytopenia

**DOI:** 10.3390/v17070950

**Published:** 2025-07-04

**Authors:** Martha A. Mendoza-Hernandez, Janet Diaz-Martinez, Gustavo A. Hernández-Fuentes, Fabian Rojas-Larios, Katya A. Cárdenas-Cárdenas, Paulina García de León-Flores, David A. Rojas-Cruz, Roberto Aceves-Calvario, Ernesto Gómez-Sandoval, Montserrat Árciga-García, José Guzmán-Esquivel, Valery Melnikov, Francisco Espinoza-Gómez, Iván Delgado-Enciso

**Affiliations:** 1 Hospital General de Manzanillo IMSS-Bienestar, Av. Elías Zamora Verduzco S/N, Nuevo Salagua, Manzanillo City 28869, Mexico; mendoza_martha@ucol.mx (M.A.M.-H.); katyacardenas12@gmail.com (K.A.C.-C.); pau.g96@hotmail.com (P.G.d.L.-F.); drojas3@ucol.mx (D.A.R.-C.); roberto_aceves0@ucol.mx (R.A.-C.); gosaer480@icloud.com (E.G.-S.); rabe_m21@hotmail.com (M.Á.-G.); 2 Department of Molecular Medicine, School of Medicine, University of Colima, Colima City 28040, Mexico; gahfuentes@gmail.com (G.A.H.-F.); frojas@ucol.mx (F.R.-L.); valery.melnikoff@gmail.com (V.M.); 3 Research Center in Minority Institutions, Department of Dietetics and Nutrition, Florida International University (FIU-RCMI), Miami, FL 33199, USA; jdimarti@fiu.edu; 4 Faculty of Chemical Sciences, University of Colima, Coquimatlan City 28400, Mexico; 5 State Cancerology Institute of Colima, Health Services of the Mexican Social Security Institute for Welfare (IMSS-BIENESTAR), Colima City 28085, Mexico; 6 Clinical Epidemiology Research Unit, Mexican Institute of Social Security, Villa de Alvarez 28984, Mexico; jose.esquivel@imss.gob.mx; 7 Regional University Hospital (IMSS-Bienestar), Health Services of the State of Colima, Colima City 28060, Mexico; fespin@ucol.mx; 8 Robert Stempel College of Public Health and Social Work, Florida International University, Miami, FL 33199, USA

**Keywords:** dengue virus serotype 3 (DENV-3), neurological complications, renal dysfunction, polyserositis, epidemiology

## Abstract

Dengue mortality has traditionally been associated with severe thrombocytopenia and hemorrhagic complications. However, during 2024, dengue virus serotype 3 (DENV-3) increased significantly in western Mexico, leading to the emergence of a distinct clinical pattern. We conducted a retrospective cohort study of hospitalized dengue patients at the General Hospital of Colima (January–August 2024). Clinical features, laboratory parameters, and outcomes were compared between survivors and non-survivors. Among 201 hospitalized patients, 6 (3.0%) died. All deceased patients presented with generalized seizures, polyserositis (pleural effusion and/or ascites), and required mechanical ventilation. Contrary to classical patterns, they did not have severe thrombocytopenia. Instead, they showed significantly higher white blood cell counts and notably increased levels of serum urea and BUN, suggesting early renal impairment. ROC analysis indicated that BUN (AUC 0.904) and urea (AUC 0.906) were good to excellent discriminators of mortality. During 2024, with an increase in DENV-3 circulation, mortality was associated with neurological and systemic complications, including seizures and polyserositis, as well as biochemical evidence of renal dysfunction—but not with severe thrombocytopenia. These findings challenge current paradigms and highlight the need for early recognition of atypical clinical patterns.

## 1. Introduction

Dengue virus infection is a major and growing public health concern worldwide, with an estimated 390 million infections and 25,000 deaths annually, predominantly in tropical and subtropical regions [1,2]. In the Americas, the burden of dengue reached record levels in 2024, with Mexico experiencing one of the most severe epidemics in its history [3]. Notably, the state of Colima has emerged as one of the most affected regions in Mexico, reporting the highest national incidence rate—over 622 cases per 100,000 inhabitants in 2024—amid a dramatic surge in cases [4]. This epidemiological scenario has placed unprecedented strain on local healthcare systems and highlighted the urgent need for improved clinical management and public health strategies.

Historically, severe dengue has been linked to plasma leakage and hemorrhagic complications, primarily driven by severe thrombocytopenia [5]. Consistently, the WHO classification highlights warning signs such as persistent vomiting, mucosal bleeding, and a rapid drop in platelet count as predictors of severe disease [6,7]. However, recent outbreaks have challenged these classical paradigms. Emerging evidence suggests that the clinical spectrum of severe dengue is broader and more heterogeneous than previously recognized, with increasing reports of atypical presentations—including neurological involvement, acute organ dysfunction, and systemic inflammatory responses—particularly in adults and patients with comorbidities [8].

The 2024 epidemic in Mexico has been characterized by a predominance of dengue virus serotype 3 (DENV-3) [9], a strain historically associated with more severe disease manifestations and increased risk of outbreaks in previously exposed populations [10]. Furthermore, the interplay between host factors (such as comorbidities and immune status), viral characteristics, and environmental determinants is increasingly recognized as central to understanding the pathogenesis and outcomes of dengue infection [11]. Recent studies have highlighted the potential prognostic value of laboratory markers beyond thrombocytopenia, including leukocyte indices, markers of renal function, and inflammatory ratios, in identifying patients at highest risk of adverse outcomes [12,13,14].

Against this backdrop, there is a pressing need to refine risk stratification tools and clinical management algorithms for dengue, particularly in high-incidence settings. A deeper understanding of the constantly evolving clinical and biochemical predictors of mortality is essential to optimize patient outcomes, efficiently allocate resources, and inform public health policies during epidemic emergencies. Therefore, this paper reports the clinical outcomes of hospitalized patients in Colima, Mexico, emphasizing the signs and parameters of those who died.

## 2. Materials and Methods

### 2.1. Study Design

A retrospective observational study was carried out using data from a cohort of patients hospitalized with confirmed DENV-3 infection during the 2024 epidemic in a tropical/coastal city in the state of Colima, Mexico. The study was conducted at the General Hospital of Manzanillo IMSS-Bienestar “Hospital General de Manzanillo IMSS-Bienestar” as part of a project that analyzes the epidemiology of dengue over time. It was approved by the Central HRU (Regional University Hospital of Colima “Hospital Regional Universitario de Colima”) Research Ethics Committee (approval code: CI 2024/2/SR/MI/254). This study adhered to the Strengthening the Reporting of Observational Studies in Epidemiology (STROBE) guidelines. As it was a retrospective analysis based on medical records, the ethics committee waived the requirement for informed consent [15]. Nonetheless, strict measures were taken to ensure the anonymity and confidentiality of all patient data.

Inclusion criteria comprised (1) age ≥ 16 years; (2) laboratory-confirmed dengue infection, defined as positive NS1 antigen and/or IgM/IgG serology and/or PCR, according to national guidelines; and (3) hospitalization for clinical management. Patients with incomplete clinical records or who were discharged against medical advice were excluded.

### 2.2. Data Collection and Outcomes

Data were extracted manually from electronic medical records and archived laboratory databases by two independent investigators using a structured case report form. The primary outcome was in-hospital mortality, defined as death occurring during hospitalization due to any cause. Patients were stratified post hoc into survivors and deceased for comparative analyses. Others variables were collected for analysis: demographic and comorbidity data, including age, sex, and the presence of diabetes mellitus, hypertension, obesity, smoking status, autoimmune diseases, HIV infection, ischemic heart or cerebrovascular disease, and chronic kidney disease; pharmacological treatments administered during hospitalization, including the use of paracetamol, antihypertensive agents, insulin, corticosteroids, non-steroidal anti-inflammatory drugs (NSAIDs), and antibiotics; and laboratory parameters obtained during hospitalization.

Hematological parameters included hemoglobin, hematocrit, red cell distribution width (RDW), leukocyte count, and differential counts (neutrophils, lymphocytes, monocytes, eosinophils, and basophils). Absolute white blood cell counts (neutrophils, lymphocytes, monocytes, eosinophils, and basophils) were reported in number of cells per microliter (×10^3^/μL), as determined by an automated hematology analyzer (DxH 690T, Beckman Coulter, Brea, CA, USA). These values represent the actual cell count, in contrast to relative percentages, and allow for a more accurate assessment in the context of leukopenia, which is common in dengue infection [16,17].

Biochemical analyses were performed using a fully automated dry chemistry analyzer (VITROS 5600 Integrated System, Ortho Clinical Diagnostics, Rochester, NY, USA), and included serum glucose, blood urea nitrogen (BUN), urea, creatinine, total, direct, and indirect bilirubin, and liver enzymes including aspartate aminotransferase (AST), alanine aminotransferase (ALT), gamma-glutamyl transferase (GGT), alkaline phosphatase (ALP), lactate dehydrogenase (LDH), and serum albumin. Liver function tests and biochemical panels were also processed using the VITROS 5600 platform. Coagulation parameters included prothrombin time (PT), activated partial thromboplastin time (aPTT), and international normalized ratio (INR), and were assessed using an automated coagulometer (ACL TOP 550 CTS, Instrumentation Laboratory, Bedford, MA, USA).

All laboratory analyses were performed at the clinical laboratory of Hospital of Manzanillo IMSS-Bienestar, located in Colima, Mexico, and were conducted under standardized protocols and internal quality controls.

### 2.3. Statistical Analysis

Continuous variables were tested for normality using the Shapiro–Wilk test. Since most variables displayed non-normal distribution, they are reported as medians with 25th and 75th percentiles (Q1 and Q2). Categorical variables are presented as frequencies and percentages. Comparisons between survivors and non-survivors were made using the Mann–Whitney U test for continuous variables and with Fisher’s exact test for categorical variables. Receiver operating characteristic (ROC) curve analysis was used to evaluate the discriminative ability of selected clinical and biochemical parameters in predicting in-hospital mortality among patients with dengue. All evaluated variables were included in this analysis to comprehensively explore their potential prognostic value. Although not all parameters reached statistical significance, they are retained in the results to provide a complete profile and to facilitate future research [18,19].

Given the small sample size, we opted not to report sensitivity, specificity, or optimal cutoff points, as these values may lack robustness and clinical reliability and lead to overfitting and misleading conclusions. Instead, we focused on reporting the area under the curve (AUC) with corresponding 95% confidence intervals (CIs) to explore the potential utility of these markers as prognostic indicators. AUC values were interpreted as follows: >0.90 = excellent, 0.80–0.89 = good, between 0.70 and 0.79 as fair, and between 0.50 and 0.69 as poor [20]. The aim was to preliminarily assess whether these variables show potential discriminatory value, as reflected by the AUC [21]. A *p*-value < 0.05 was considered statistically significant. Analyses were performed using SPSS Statistics version 20 software program (IBM Corp., Armonk, NY, USA) [22].

### 2.4. Quality Control

Double data entry and verification were employed to minimize transcription errors. Inconsistencies were resolved through review by a third investigator. Missing data were handled by pairwise deletion and not imputed, due to the retrospective design and low frequency of missingness.

## 3. Results

A total of 196 patients hospitalized with laboratory-confirmed dengue infection were included in the analysis, of whom 6 (3.1%) died during hospitalization. The minimum and maximum ages were 16 and 87 years, respectively. Table 1 summarizes the main demographic and clinical characteristics of the patients, stratified by vital status at hospital discharge. Patients who died were significantly older than those who survived (median age 53 [IQR 28–65] vs. 27 years [18,19,20,21,22,23,24,25,26,27,28,29,30,31,32,33,34,35,36,37,38,39]; *p* = 0.036). Most patients who died were men, although a significant difference was not reached (83.3% vs. 49.7, *p* = 0.212). Comorbidities such as diabetes mellitus (50.0% vs. 10.0%; *p* = 0.020) and hypertension (50.0% vs. 7.2%; *p* = 0.008) were significantly more prevalent among those who died. Obesity, smoking, autoimmune diseases, and chronic kidney disease showed no significant differences between groups. Regarding pharmacological management, paracetamol was administered to 100% of deceased patients and 65.6% of survivors (*p* = 0.182). Notably, antihypertensive drugs were more frequently used in patients who died (33.3% vs. 5.2%; *p* = 0.045), consistent with their higher burden of cardiovascular comorbidities.

### 3.1. Laboratory Findings Prior to Discharge

Table 2 compares hematological and biochemical parameters between survivors and deceased patients, focusing on values obtained during the final two days of hospitalization. Deceased patients had significantly lower hemoglobin levels (median 9.95 g/dL [8.28–11.75] vs. 13.80 g/dL [12.70–15.10]; *p* < 0.001) and hematocrit (27.95% [23.63–32.63] vs. 39.30% [36.50–42.30]; *p* < 0.001). Red cell distribution width (RDW) was significantly higher among deceased patients (*p* = 0.007), suggesting greater erythrocyte anisocytosis.

Unexpectedly, platelet counts were significantly higher in the deceased group (median 94.5 × 10^3^/μL [48.75–115.50] vs. 50.0 × 10^3^/μL [35.00–66.00]; *p* < 0.001).

Markers of renal function, including blood urea nitrogen (BUN), urea, and creatinine, were significantly worse in deceased patients (all *p* < 0.001), suggesting that acute kidney injury may have contributed to mortality. Serum albumin levels were also significantly lower in this group (median 2.00 g/dL vs. 3.30 g/dL; *p* < 0.001), consistent with hypoalbuminemia, which is associated with systemic inflammation and capillary leakage.

Although transaminase levels (AST and ALT) were elevated in both groups, they were paradoxically lower in deceased patients (*p* = 0.001 and *p* < 0.001, respectively), possibly reflecting hepatic exhaustion or variability in sampling timing during the terminal phase of illness.

Coagulation parameters, including prothrombin time (PT), international normalized ratio (INR), and activated partial thromboplastin time (aPTT), showed statistically significant differences between groups; however, these changes did not appear to be clinically relevant.

### 3.2. Blood Markers with Potential Predictive Value for Mortality

A receiver operating characteristic (ROC) curve analysis was conducted to evaluate the ability of various clinical and biochemical parameters to discriminate mortality among hospitalized patients with dengue. Several markers showed good to excellent discriminatory capacity, particularly urea (AUC: 0.906; 95% CI: 0.808–1.000; *p* < 0.001), blood urea, and nitrogen (BUN; AUC: 0.904; 95% CI: 0.805–1.000; *p* < 0.001). Other parameters, such as platelet count and absolute neutrophil count, demonstrated moderate predictive performance. The full results are summarized in Table 3.

### 3.3. Key Clinical Differences Between Survivors and Non-Survivors

Notable differences in clinical presentation were observed between patients who survived and those who died during hospitalization for dengue. All non-survivors (6/6; 100%) required invasive mechanical ventilation (IMV) and experienced seizures, while none of the survivors (0/190; 0%) had these complications (*p* < 0.001 for both). Likewise, polyserositis—assessed in a subset of patients who underwent thoracoabdominal ultrasound (*n* = 135)—was present only among those who died (6/6; 100%) and absent in survivors (0/129; 0%) (*p* < 0.001). No significant difference was found in the presence of hepatomegaly between the two groups (1/129 survivors, 0.8%; 0/6 non-survivors, 0%; *p* = 0.956). These findings outline clinical severity in fatal cases. Comparisons were performed using Fisher’s exact test.

## 4. Discussion

Currently, Mexico, as well as several Latin American countries are facing a significant dengue outbreak, with some regions reporting record-breaking numbers of cases. In the last decade, DENV-2 predominated in Mexico, but in 2023 and especially 2024, the prevalence of DENV-3 cases increased considerably, leading to the emergence of atypical clinical presentations, with greater liver involvement, as well as respiratory symptoms that simulate COVID-19 or other respiratory viruses, with normal or slightly decreased platelet counts [23]. However, this is an underreported phenomenon. During 2024, the Colima region had the highest incidence of confirmed dengue cases in Mexico (622 confirmed cases per 100,000 inhabitants) [4]. The 2024 outbreak in Colima, Mexico, has unveiled an atypical clinical and biochemical profile associated with in-hospital mortality, prompting critical reconsideration of existing paradigms for dengue severity assessment. Typically, severe thrombocytopenia and hemorrhagic manifestations have been considered hallmark predictors of poor outcomes in dengue fever, particularly in previous studies [24]. However, the findings of the present study demonstrate that during this outbreak, mortality was not linked to classical hemorrhagic complications or profound thrombocytopenia. Instead, neurological events (notably generalized seizures), systemic inflammatory features (such as polyserositis), and early renal dysfunction emerged as the most salient predictors of mortality.

Among the six patients who died, they were mostly men, were significantly older than the survivors (median age 53 vs. 27 years), and exhibited a higher prevalence of diabetes mellitus and hypertension. The gender difference in mortality, although not statistically significant, is consistent with previous reports showing higher mortality in men [25]. Nonetheless, prior studies have highlighted that age and comorbidities modulate the host immune response, potentially exacerbating dengue pathophysiology. A multicenter study in Mexico [26] also reported that patients with diabetes and hypertension were more likely to develop severe dengue and succumb to complications [27], possibly due to chronic endothelial dysfunction and impaired immune regulation. Similar associations were observed during DENV-2 and DENV-1 outbreaks in other regions of America [28,29,30]. Diabetes mellitus has been implicated in amplifying dengue-associated plasma leakage and cytokine storms [31,32,33]. Hyperglycemia is known to upregulate pro-inflammatory mediators and compromise leukocyte function, leading to an exaggerated yet ineffective immune response [34]. Hypertension, on the other hand, may predispose microvascular damage and exacerbate capillary leakage, culminating in multiorgan dysfunction [35].

Contrary to long-standing beliefs that low platelet counts are central to dengue mortality, our data demonstrate that non-survivors exhibited significantly higher platelet counts (median 94.5 × 10^3^/μL) than survivors (median 50.0 × 10^3^/μL). This unexpected s inversion may reflect distinct immunopathological dynamics associated with DENV-3 circulation in this region. Historically, outbreaks caused by DENV-2 and DENV-1 have shown stronger associations with platelet consumption, disseminated intravascular coagulation (DIC), and bleeding [36,37]. In contrast, DENV-3, particularly certain genotypes, has demonstrated a higher neurotropic potential in animal models [38], possibly indicating a different pathophysiological profile. However, this finding should be interpreted with caution due to the limited sample size of fatal cases and the single-center design of our study. It is possible that regional factors, timing of sample collection, or viral genotypic variations influenced the observed results; for that reason, more studies are needed with broader populations and different epidemic contexts.

This neurotropism has also been reported in patients, primarily with DENV-2 and DENV-3 infections [39], although cases involving DENV-1 have also been documented [33]. The relatively preserved platelet counts observed in non-survivors may indicate a distinct pathophysiological pathway in which mortality is driven not by hemorrhagic complications but rather by inflammatory and organ-specific mechanisms—particularly those affecting the brain, lungs, and kidneys. Notably, none of the deceased exhibited hemorrhagic symptoms.

All patients who died developed generalized seizures, a feature not typically emphasized in standard dengue severity classifications [40]. Although neurological manifestations of dengue are increasingly recognized—including encephalopathy, encephalitis, and seizures—they remain underreported in most clinical settings [41]. A study from India has proposed that neuro-dengue can occur due to (1) direct neurotropism (encephalitis, meningitis, myelitis, and myositis); (2) systemic complications (encephalopathy, stroke, and hypokalemic paralysis); (3) post-infectious/immune-mediated mechanisms (acute disseminated encephalomyelitis (ADEM), Guillain–Barré syndrome, and optic neuritis) [42,43]. The universal presence of seizures in all fatal cases in the present report suggests a profound neurotropic effect or secondary neuroinflammation, possibly exacerbated by renal dysfunction and uremic encephalopathy. The elevated BUN and urea levels in these patients support this hypothesis. While cerebrospinal fluid (CSF) data were unavailable, the clinical constellation aligns with previous descriptions of dengue-associated encephalopathy and post-infectious neuroinflammation. DENV-3 has previously been shown to be one of the dengue virus types with the highest neurotropism, especially certain variants [44,45]. In the region where the present study was conducted, DENV-1, -2, and -3 were circulating. It is plausible that DENV-3 in this area may exhibit greater neurovirulence; however, the possibility that DENV-1 or DENV-2 may be responsible cannot be ruled out [46], highlighting the need for further specific investigation.

Polyserositis (presence of pleural effusion and/or ascites) was observed in all patients who died, consistent with capillary leak syndrome. However, these findings occurred in the absence of classical hemoconcentration, as indicated by lower hematocrit levels in non-survivors. This discrepancy points to a severe hypoalbuminemic state, as confirmed by significantly reduced serum albumin levels (median 2.00 g/dL), which impairs intravascular oncotic pressure and promotes third-spacing. Capillary leakage without hemoconcentration suggests an atypical presentation, possibly involving a more aggressive or dysregulated inflammatory response, or it may reflect a secondary effect of treatment—particularly the administration of intravenous fluids [47,48]. Hypoalbuminemia may also reflect malnutrition, systemic capillary leak, or hepatic dysfunction [49,50]. Interestingly, AST and ALT levels were lower in non-survivors than in survivors, which may suggest that liver involvement was not a determining factor in the fatal outcomes of these patients, contrary to what has been reported in previous studies [43].

Among all biochemical parameters, BUN (AUC 0.91) and urea (AUC 0.89) demonstrated the highest discriminative power for mortality. Creatinine levels were also elevated, albeit with a smaller magnitude. These findings suggest acute kidney injury (AKI) as a central contributor to dengue mortality in this cohort, despite the absence of pre-existing chronic kidney disease. Renal impairment in dengue has often been considered a terminal event or consequence of severe plasma leakage [44,45,46]. However, emerging evidence suggests that AKI may develop early due to direct viral injury, systemic inflammation, or renal hypoperfusion [44,45,46]. Moreover, uremia may contribute to encephalopathy and seizures, as discussed earlier, forming a vicious cycle. Although MPV was not significantly different between groups in our cohort, we included it due to prior studies suggesting its relevance in inflammatory and infectious conditions, including dengue. Its inclusion may guide future analyses of hematologic indicators associated with disease severity [51].

High RDW values in fatal cases (13.2% vs. 12.7%) may indicate ineffective erythropoiesis or oxidative stress-induced hemolysis, both linked to systemic inflammation. An increased red cell distribution width (RDW) has been linked to higher levels of pro-inflammatory cytokines, including tumor necrosis factor alpha and interleukin-6. These inflammatory mediators reduce the effectiveness of erythropoietin and promote the release of immature red blood cells into the circulation, which contributes to the elevation of RDW [47,48]. While these markers are non-specific, their consistent elevation in the fatal group suggests that dengue-related deaths may result from an uncontrolled immune cascade, as has been reported in other diseases such as COVID-19 [49], rather than isolated organ failure. All deceased patients received paracetamol, and half had comorbid diabetes and hypertension, with increased use of antihypertensives. While NSAIDs and steroids were not administered (appropriately, per guidelines), the high burden of systemic inflammation observed calls into question whether anti-inflammatory adjuncts, such as corticosteroids or IL-6 inhibitors, might be beneficial in a subset of patients with hyperinflammatory profiles. A previous study by Espinoza-Gómez (2017) [50] suggested that the administration of methylprednisolone may be beneficial in managing patients with dengue presenting warning signs [50]. However, a systematic review of randomized trials in dengue has so far shown no clear benefit from the use of corticosteroids [52].

This study has certain limitations that should be acknowledged. First, it was conducted at a single referral center in Colima, Mexico, which may limit the generalizability of the findings to other regions with different circulating DENV serotypes or healthcare settings. Second, although several clinically relevant differences were identified between survivors and non-survivors, the limited number of fatal outcomes available for analysis constrains the statistical power for some comparisons. To address this, we applied appropriate non-parametric statistical methods to ensure the robustness of our results. Nonetheless, expanding the study population through multicenter collaborations or incorporating data from other regions could allow for a broader representation of disease severity, improve the external validity of the findings, or enable the detection of other potential biomarkers. It is important to acknowledge that the clinical heterogeneity observed may also be influenced by previous exposures to other dengue virus serotypes which were not assessed in this study. The phenomenon of immune imprinting or antibody-dependent enhancement (ADE) could contribute to the severity of disease manifestations, especially in secondary infections, and may partly explain the atypical laboratory and clinical profiles observed in our non-survivor group [53]. Incorporating serological or immunological data in future research will be essential to disentangle these effects.

Third, its retrospective design restricts the ability to infer causality, and certain advanced diagnostics—such as cerebrospinal fluid analysis, viral genotyping, or imaging studies—were not uniformly available. These tools, while not essential for the initial objectives of this study, represent important opportunities for future research aiming to further elucidate the mechanisms underlying neurological and renal involvement. Finally, the absence of cytokine profiling and other inflammatory markers limits immunopathological insight yet highlights the need to incorporate these variables in subsequent, more comprehensive studies. Despite these limitations, the findings contribute valuable clinical observations that can inform early recognition and management strategies in severe dengue cases.

This study is based on a single-center retrospective study with a small number of fatal outcomes. Despite this, the consistent clinical and biochemical patterns observed among non-survivors highlight biologically plausible and clinically relevant phenomena. Prospective validation in larger, multicenter cohorts is essential. Furthermore, viral genotyping, cytokine profiling, and imaging studies (e.g., brain MRI, renal ultrasound) could clarify the mechanisms of neuro-renal involvement. Finally, updated clinical scores incorporating BUN, seizures, and albumin levels may provide more accurate triage tools during DENV-3 outbreaks, particularly when classical warning signs (like thrombocytopenia) are absent. The need for mechanical ventilation in all fatal cases also underscores the importance of early recognition and referral. In resource-limited settings, delays in critical care access may worsen outcomes, particularly for neurological and respiratory complications.

## 5. Conclusions

In conclusion, the 2024 DENV outbreak in western Mexico (with increasing presence of DENV-3) revealed a lethal clinical phenotype characterized by neurological involvement, polyserositis, systemic inflammation, and early renal dysfunction, in the absence of severe thrombocytopenia. These findings challenge traditional assumptions about dengue mortality and underscore the heterogeneity of dengue pathophysiology across serotypes and populations. Timely recognition of atypical patterns, especially in older adults with comorbidities, is critical to reducing dengue-related deaths. A paradigm shift is urgently needed, both in clinical training and in WHO severity classifications, to include non-hemorrhagic markers of poor prognosis in dengue.

## Figures and Tables

**Table 1:** Baseline characteristics and medications administered to hospitalized patients with dengue, stratified by vital status at discharge.

Variable	Survivors (*n* = 190)	Deceased (*n* = 6)	*p*-Value
Age (years)	27 (20–41)	53 (28–65)	0.036
Female	50.3%	16.7%	0.212
Obesity	7.2%	0.0%	0.645
Diabetes mellitus (DM)	10.0%	50.0%	0.020
Hypertension (HTN)	7.2%	50.0%	0.008
Smoking	11.0%	16.7%	0.513
Pulmonary disease	1.0%	0.0%	0.945
Autoimmune disease	0.5%	0.0%	0.972
HIV	1.4%	0.0%	0.921
Ischemic disease	0.5%	0.0%	0.972
Chronic kidney disease	1.0%	0.0%	0.945
IgG positive	82.5%	80.0%	0.999
Medications Administered			
Paracetamol	65.6%	100.0%	0.182
Antibiotics	1.4%	0.0%	0.918
NSAIDs	0.0%	0.0%	
Steroids	1.0%	0.0%	0.999
Antihypertensives	5.2%	33.3%	0.045
Insulin	1.6%	0.0%	0.911

A comparison between groups was performed using Fisher’s exact test for categorical variables. Age is presented as the median (25th and 75th percentiles) and was compared using a one-tailed Mann–Whitney U test, under the hypothesis that “those who died were older than those who survived”.

**Table 2:** Comparison of hematological and biochemical parameters between survivors and deceased patients with dengue during final two days of hospitalization.

	Survivors			Non-Survivors			*p*-Value
Parameter	Median	Q1	Q3	Median	Q1	Q3	
Hemoglobin (g/dL)	13.80	12.70	15.10	9.95	8.28	11.75	<0.001
Hematocrit (%)	39.30	36.50	42.30	27.95	23.63	32.63	<0.001
RDW-CV (%)	12.70	12.10	13.50	13.20	12.70	14.00	0.007
Platelets (×103/μL)	50.00	35.00	66.00	94.50	48.75	115.50	<0.001
Mean Platelet Volume (MPV, fL)	11.85	11.30	12.60	12.70	11.08	13.20	0.575
White Blood Cells (×103/μL)	5.07	3.81	6.94	9.77	4.55	13.29	<0.001
Absolute Neutrophils (×103/μL)	1.57	1.00	2.43	6.99	2.61	11.17	<0.001
Absolute Lymphocytes (×103/μL)	2.60	1.90	3.49	1.25	1.11	1.74	<0.001
Absolute Monocytes (×103/μL)	0.46	0.34	0.63	0.54	0.32	0.98	<0.001
Absolute Eosinophils (×103/μL)	0.05	0.02	0.11	0.07	0.01	0.15	0.227
Absolute Basophils (×103/μL)	0.04	0.03	0.06	0.03	0.01	0.04	0.122
Glucose (mg/dL)	91.00	84.50	106.00	96.00	84.00	123.00	0.004
BUN (mg/dL)	7.00	6.00	10.00	16.00	12.75	62.50	<0.001
Urea (mg/dL)	14.98	12.84	21.40	34.24	27.29	133.71	<0.001
Creatinine (mg/dL)	0.70	0.60	0.80	0.80	0.60	1.50	<0.001
Direct Bilirubin (mg/dL)	0.38	0.29	0.50	0.40	0.21	2.96	0.163
Indirect Bilirubin (mg/dL)	0.29	0.18	0.41	0.29	0.13	0.38	0.922
Total Bilirubin (mg/dL)	0.68	0.50	0.86	0.69	0.34	3.32	0.164
AST (U/L)	234.00	135.50	387.50	125.50	74.75	271.75	0.001
ALT (U/L)	156.00	90.75	250.00	75.50	31.00	219.75	<0.001
GGT (U/L)	209.00	98.50	359.00	102.50	42.00	308.75	0.078
Alkaline Phosphatase (U/L)	95.00	65.00	141.00	149.50	38.25	243.50	0.638
LDH (U/L)	529.50	404.75	848.50	551.00	322.00	1180.50	0.072
Albumin (g/dL)	3.30	3.00	3.60	2.00	1.85	2.05	<0.001
Prothrombin Time (PT, s)	11.00	10.50	11.70	12.50	11.30	12.43	0.011
aPTT (s)	36.70	33.00	42.00	30.60	28.00	32.75	<0.001
INR	0.95	0.90	1.01	1.08	0.97	1.19	0.010

Data are shown as the median (Q1–Q3). All comparisons made using the non-parametric Mann–Whitney U test. BUN: blood urea nitrogen; AST: aspartate aminotransferase; ALT: alanine aminotransferase; GGT: gamma-glutamyl transferase; PT: prothrombin time; aPTT: activated partial thromboplastin time; INR: international normalized ratio.

**Table 3:** Discriminatory capacity (AUC) of clinical and laboratory parameters for mortality prediction in patients hospitalized with dengue.

		CI 95%		*p*-Value
Parameter	AUC	Lower	Upper	
Hemoglobin (HG)	0.141	0.000	0.303	<0.001
Hematocrit (HTO)	0.129	0.000	0.290	<0.001
RDW-CV	0.646	0.519	0.772	0.100
Platelets	0.799	0.623	0.976	0.001
Mean Platelet Volume (MPV)	0.638	0.421	0.855	0.136
Leukocytes	0.757	0.576	0.939	0.006
Absolute Neutrophils	0.835	0.671	0.998	<0.001
Absolute Lymphocytes	0.166	0.060	0.271	<0.001
Absolute Monocytes	0.605	0.379	0.831	0.259
Absolute Eosinophils	0.571	0.374	0.769	0.442
Absolute Basophils	0.353	0.159	0.547	0.113
Glucose	0.519	0.313	0.726	0.835
Blood Urea Nitrogen (BUN)	0.904	0.805	1.000	<0.001
Urea	0.906	0.808	1.000	<0.001
Creatinine	0.624	0.427	0.822	0.180
Direct Bilirubin (DB)	0.547	0.249	0.846	0.691
Indirect Bilirubin (IB)	0.448	0.236	0.661	0.664
Total Bilirubin (TB)	0.528	0.217	0.839	0.815
AST (TGO)	0.266	0.105	0.428	0.002
ALT (TGP)	0.237	0.085	0.389	0.001
GGT	0.418	0.298	0.538	0.279
Alkaline Phosphatase (ALP)	0.571	0.383	0.760	0.346
LDH	0.359	0.228	0.490	0.063
Albumin	0.106	0.000	0.249	<0.001
Prothrombin Time (PT)	0.767	0.663	0.870	0.010
aPTT	0.066	0.014	0.118	<0.001
INR	0.767	0.648	0.882	0.010

The area under the curve (AUC), 95% confidence intervals (CIs), and *p*-values are presented for each variable. Due to the limited sample size, optimal cutoff points, sensitivity, and specificity values were not included to avoid potential overestimation.

## Data Availability

The original contributions presented in this study are included in the article; further inquiries can be directed to the corresponding author.
